# The emperor has no clothes: OAB can be cured surgically

**DOI:** 10.1007/s00192-022-05130-8

**Published:** 2022-03-10

**Authors:** Peter Petros

**Affiliations:** University of Western Australia School of Engineering and Mathematical Sciences, WA Perth, Australia

**Keywords:** Overactive bladder, Urge, Frequency, Nocturia, Surgical cure, Uterosacral ligaments, Cardinal ligaments

## Abstract

The paper by Karjalainen et al., who reviewed 2,933 pelvic organ prolapse surgeries, showed 75% cure for “bothersome” urge urinary incontinence (UUI), is more than an “Aha” moment; it is an “Emperor has no clothes” moment. Since 1976, a convention of “no surgery” for women with UUI (now overactive bladder, OAB) has become almost an article of faith. Yet, surgical cure of OAB has been known since 1997, when this journal published the first urodynamically controlled study with 20-month data: 86% cure for UUI, 85% for frequency, 80% for nocturia following pubourethral ligament (PUL) and uterosacral ligament (USL) sling repair in 85 women. This study was followed by many other publications over the years recording OAB cure. It is not that even a small fraction of the 600 million women on the planet will now undergo surgery, or that damaged ligaments are the only cause of OAB. However, knowing OAB can be cured opens the door for young creative minds to bring hope and relief to these women non-surgically, as well as surgically.

Some 20–30% of women on the planet suffer from overactive bladder (OAB) and up to 50% of 80-year-old women suffer from nocturia. Human suffering and broken hips aside, some experts put the financial cost to the community at up to USD 61 billion per annum.

Since the first ICS consultation in 1976 [1] until now, learned bodies [2] have consistently advised that the pathogenesis of OAB, (urge, frequency, nocturia) was unknown [2], that no cure was possible and that surgery was contraindicated. Such comments appear to have become an article of faith, a convention that has continued despite many publications since 1976 stating otherwise.

The paper by Karjalainen et al. [3], who reviewed 2,933 pelvic organ prolapse surgeries, showed 75% cure for “bothersome” urge urinary incontinence (UUI), is more than an “Aha” moment; it is an “Emperor has no clothes” moment, a moment when the convention is forever broken.

Twenty-five years ago, in 1997, this journal published, for the first time in the literature, a study describing the surgical cure of multiple bladder symptoms following day-care pubourethral ligament (PUL) and uterosacral ligament (USL) sling repair [4]. The study was pre- and post-operatively urodynamically controlled. The following cure rates were reported: stress incontinence (88%), UUI (86%), frequency (85%) and nocturia (80%). The study comprised 85 women with 20-month data. The methodology was prospective, with comprehensive pre- and post-operative assessment: symptoms (using a validated questionnaire), transperineal ultrasound, urodynamic tests for detrusor overactivity, flow rate, emptying time, residual urine, urethral pressure profile, cough and Valsalva transmission ratios, and pad tests. The cure rates achieved for OAB symptoms [4] were similar to those reported by Karjalainen et al. [3]. The OAB cure data by connective tissue/ligament repair [4] have been repeatedly confirmed by many surgeons since 1997.

The “Emperor moment” by Karjalainen et al. [3], uncovering an outdated convention, that OAB is not surgically curable, has a major human element. The Earth’s population in the year 2000 was 6 billion, 3 billion women, of whom 20% had OAB (600,000,000). The consequences of this convention are best considered by its effect on an individual, what it means for a desperate old woman (your mother), or worse, a desperate young woman (your wife, your daughter), to be condemned literally to a life of misery with no hope of cure, when this is not so.

How can “the Emperor be dressed”? The first step for all concerned is to cease forthwith statements to the effect that OAB is not surgically curable. Next, it is important for all surgeons in the field to apprise themselves of the many works reporting OAB cure, and, more importantly, *the anatomical basis for it.* Of particular interest in this era of mesh bans is an important paper by Shkarupa et al. [5] in women with first- and second-degree prolapse repaired solely by native cardinal/uterosacral ligament repair without vaginal excision [5]. Shkarupa et al. demonstrated similar OAB cure rates to Karjalainen et al., *but only in premenopausal women*. By 18 months, the cure rates for the post-menopausal group had fallen below 20% as against a 67.5% cure for UUI and 87.5% for nocturia in the pre-menopausal group. The difference was attributed to collagen breakdown in the post-menopausal group. They advised use of posterior slings in post-menopausal women, which work by the creation of new collagen by the inserted tapes, literally a reverse tension-free vaginal tape, as in Petros [4].

The multicentre USL sling paper by Liedl et al., ”*Is overactive bladder in the female surgically curable by ligament repair?*” reports 12-month 85% OAB cure in 611 women, mean age 70 years, following posterior sling surgery. It is of interest for their discussion of the anatomical basis for their OAB cures [6]. The principles of cure discussed [6] remain valid for any method, surgical or non-surgical. As regards *available* non-sling surgery, my own experience indicates the native vaginal ligament repairs (see the videos), would give similar results to those of Karjalainen et al. [3] and Shkarupa et al. [5]: cardinal ligament repair https://youtu.be/aJDPOELZZfc; USL repair: https://www.youtube.com/watch?v=MGLdYHtqxzg

Although this editorial concerns ligament laxity as a cause of OAB symptoms, ligaments are clearly not the full story. A multitude of anatomical causes can activate an uncontrolled micturition (OAB), bladder infections, tumours, cervical fibroids and neurological lesions such as multiple sclerosis. Nor is it feasible for even a fraction of 600 million women to be surgically cured.

The study by Karjalainen et al. [3] has broken an outdated convention, that OAB is not surgically curable, given hope to women sufferers and opened the door for young creative minds to discover a plethora of innovative methods to bring hope and relief to women non-surgically, as well as surgically.
